# Supplementary material for: Single-cell analysis of the epigenome and 3D chromatin architecture in the human retina
Source: Sci Adv. 2026 Jul 23;12(30):eadv9162. doi: 10.1126/sciadv.adv9162 (PMC13394472; doi:10.1126/sciadv.adv9162)
Supplement: Supplementary file 1 — Figs. S1 to S11 Legends for tables S1 to S15 [file sciadv.adv9162_sm.pdf]

Science Advances
AAAS

Supplementary Materials for

Single-cell analysis of the epigenome and 3D chromatin architecture in the human retina

Ying Yuan et al.

Corresponding author: Radha Ayyagari, rayyagari@health.ucsd.edu; Bing Ren, br2833@cumc.columbia.edu

Sci. Adv. **12**, eadv9162 (2026)
DOI: 10.1126/sciadv.adv9162

The PDF file includes:

Figs. S1 to S11
Legends for tables S1 to S15

Other Supplementary Material for this manuscript includes the following:

Tables S1 to S15

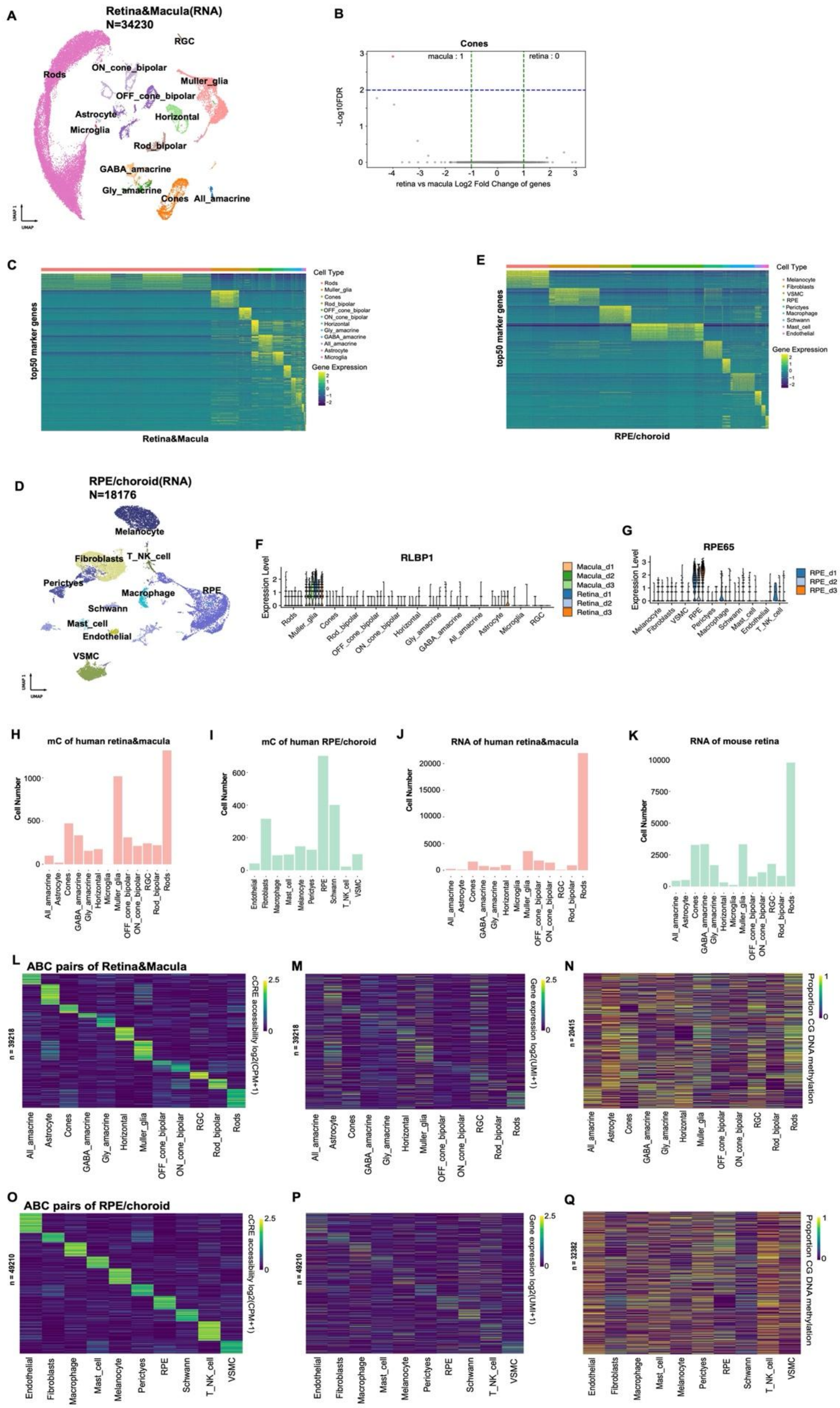
A
Retina&Macula(RNA)
N=34230
RGC
Rods
ON_cone_bipolar
Muller_glia
OFF_cone_bipolar
Astrocyte
Horizontal
Microglia
Rod_bipolar
GABA_amacrine
Gly_amacrine
Cones
All_amacrine
B
Cones
macula : 1
retina : 0
-Log10FDR
retina vs macula Log2 Fold Change of genes
C
top50 marker genes
Retina&Macula
Cell Type
Gene Expression
E
top50 marker genes
RPE/choroid
Cell Type
Gene Expression
D
RPE/choroid(RNA)
N=18176
Melanocyte
Fibroblasts
T_NK_cell
Perictyes
Macrophage
RPE
Schwann
Mast_cell
Endothelial
VSMC
F
RLBP1
Expression Level
Macula_d1
Macula_d2
Macula_d3
Retina_d1
Retina_d2
Retina_d3
G
RPE65
Expression Level
RPE_d1
RPE_d2
RPE_d3
H
mC of human retina&macula
Cell Number
I
mC of human RPE/choroid
Cell Number
J
RNA of human retina&macula
Cell Number
K
RNA of mouse retina
Cell Number
L
ABC pairs of Retina&Macula
n = 39218
cCRE accessibility log2(CPM+1)
M
n = 39218
Gene expression log2(UMI+1)
N
n = 20415
Proportion CG DNA methylation
O
ABC pairs of RPE/choroid
n = 49210
cCRE accessibility log2(CPM+1)
P
n = 49210
Gene expression log2(UMI+1)
Q
n = 32382
Proportion CG DNA methylation

Fig. S1. Single-cell multimodal analysis of human retina, macula, RPE/choroid tissues. (**A**) UMAP embedding of 10x multiome RNA-seq data with annotation for human retina and macula cell types. (**B**) Differential gene expression between Cones of human retina and macula. (**C**) Heatmap of top 50 marker gene expression levels for each cell type in retina and macula tissues. **(D)** UMAP embedding of 10x multiome RNA-seq with cell type annotation from human RPE/choroid tissue. (**E**) Heatmap of top 50 marker gene expression levels for each cell type in RPE/choroid tissue. (**F**) Marker gene of Muller glia (*RLBP1*) expression level across different donors for each cell type in retina and macula tissues. **(G)** Marker gene of RPE (*RPE65*) expression level across different donors for each cell type in RPE/choroid tissue. (**H**) Bar plot of cell numbers of each cell type of mC for human retina and macula tissues. (**I**) Bar plot of cell numbers of each cell type of mC for human RPE/choroid tissue. (**J**) Bar plot of cell numbers of each cell type of RNA for human retina and macula tissues. (**K**) Bar plot of cell numbers of each cell type of RNA for mouse retina. (**L**) Heatmap showing chromatin accessibility of cCREs for each cell type of human retina and macula tissues (the peak of peak-gene pairs predicted by ABC model). CPM, counts per million. Each CRE is ordered by the cell type with the highest accessibility level. (**M**) Heatmap showing the expression of paired genes for each cell type of human retina and macula tissues (the gene of peak-gene pairs predicted by ABC model). UMI, unique molecular identifier, here the UMI is processed with the CPM method. (**N**) Heatmap showing the DMRs corresponding to the peak of peak-gene pairs predicted for each cell type of human retina and macula tissues. (**O**) Heatmap showing chromatin accessibility of cCREs for each cell type of human RPE/choroid tissue (the peak of peak-gene pairs predicted by ABC model). (**P**) Heatmap showing the expression of paired genes for each cell type of human RPE/choroid tissue (the gene of peak-gene pairs predicted by ABC model). (**Q**) Heatmap showing the DMRs for each cell type of human RPE/choroid tissue (the DMR is corresponding to the peak of peak-gene pairs predicted by the ABC model).

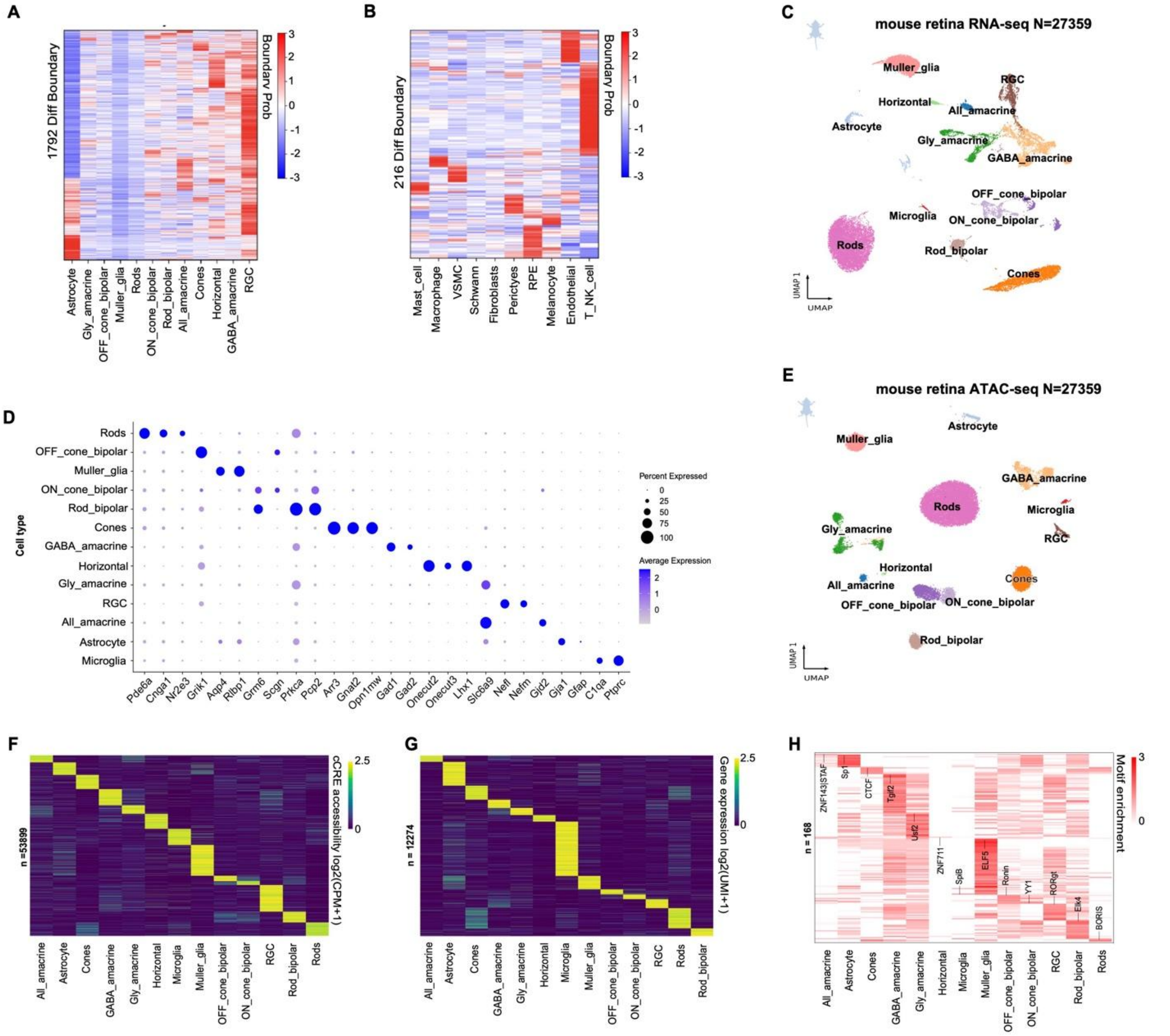

Fig. S2. Chromatin domains in human retina cell types and single cell analysis of mouse retina (**A**) Heatmaps of boundary probability of 1792 identified different TAD boundaries in each cell type of retina and macula tissues. The color bar shows the row-wise z-score normalization value of the boundary probability, red represents higher probability, blue represents lower probability. (**B**) Heatmaps of boundary probability of 216 identified different TAD boundaries in each cell type of RPE tissue. (**C**) UMAP embedding of 10x snRNA-seq data with cell type annotation for mouse retina. **(D)** Dot plot visualizing the normalized RNA expression of selected marker genes by cell type of mouse retina tissue. **(E)** UMAP embedding of 10x snATAC-seq data with cell type annotation from mouse retina. (**F**) Heatmap showing chromatin accessibility of significant cell-type specific cCREs of mouse retina. CPM, counts per million. Each CRE is ordered by the cell type with the highest accessibility level. (**G**) Heatmap showing expression of significant cell-type specific gene of mouse retina. UMI, unique molecular identifier, here the UMI is processed with the CPM method. (**H**) Enrichment of HOMER known TF motifs in cCRE regions of mouse retina.

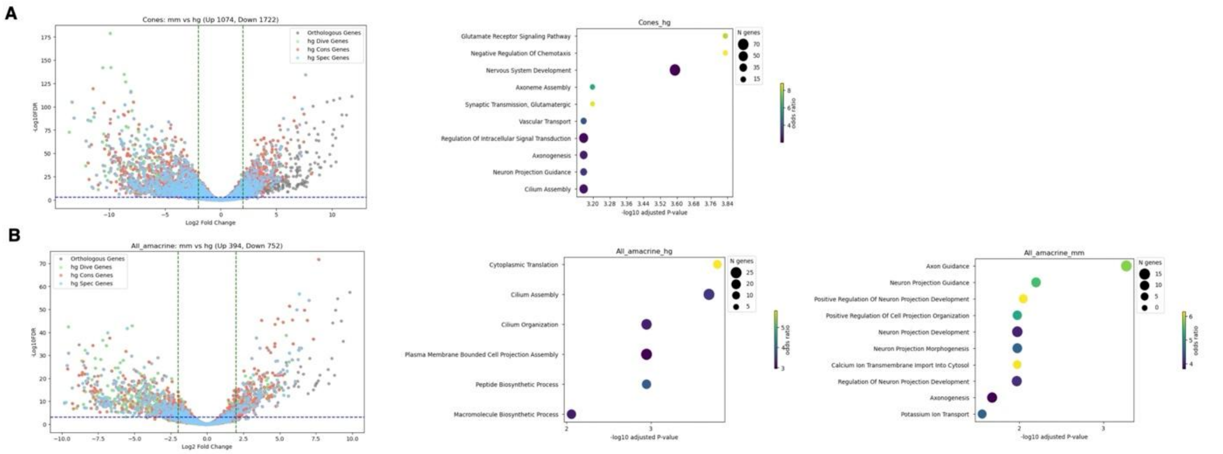

Fig. S3. Differential gene expression between human and mouse retinal cell types. (**A-B**) Volcano plot showing the differential gene expression between human and mouse Cones cells (A) or All amacrine cells (B), along with most notable GO terms associated with differentially expressed genes. Gray dots represent all the orthologous genes across human and mouse in this cell type; green dots represent the genes paired with human divergent cCREs; red dots represent the genes paired with human conserved cCREs; blue dots represent the genes paired with human specific cCREs.

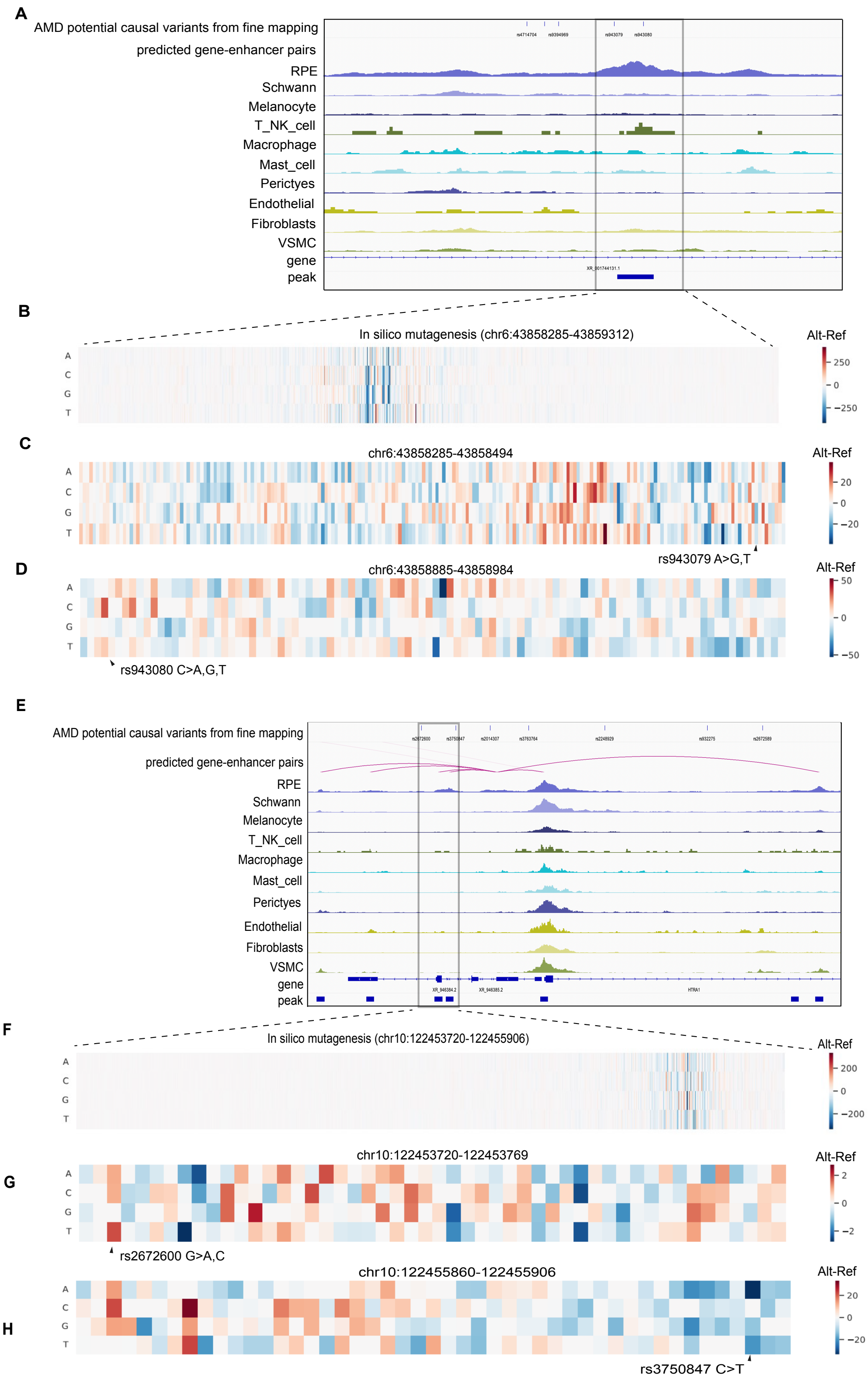

A
AMD potential causal variants from fine mapping
predicted gene-enhancer pairs
RPE
Schwann
Melanocyte
T_NK_cell
Macrophage
Mast_cell
Perictyes
Endothelial
Fibroblasts
VSMC
gene
peak
B
In silico mutagenesis (chr6:43858285-43859312)
Alt-Ref
250
0
−250
C
chr6:43858285-43858494
Alt-Ref
20
0
−20
rs943079 A>G,T
D
chr6:43858885-43858984
Alt-Ref
50
0
−50
rs943080 C>A,G,T
E
AMD potential causal variants from fine mapping
predicted gene-enhancer pairs
RPE
Schwann
Melanocyte
T_NK_cell
Macrophage
Mast_cell
Perictyes
Endothelial
Fibroblasts
VSMC
gene
peak
F
In silico mutagenesis (chr10:122453720-122455906)
Alt-Ref
200
0
−200
G
chr10:122453720-122453769
Alt-Ref
2
0
−2
rs2672600 G>A,C
H
chr10:122455860-122455906
Alt-Ref
20
0
−20
rs3750847 C>T
A
C
G
T

Fig. S4. The Deep learning model helps to interpret mutation effects on noncoding risk variants of ocular disorder and traits for the same cell type. (**A and E**) Fine mapping of AMD trait and causal risk variants in different categories of cCREs from cell types of RPE tissue. Genome browser tracks (GRCh38) display chromatin accessibility profiles from snATAC-seq, magenta arcs represent the predicted pairs of enhancer and gene from ABC model in RPE cell type. (**B**) In silico nucleotide mutagenesis influenced the prediction of accessibility within region chr6: 43858285-43859312 (0-based) of RPE cell type. Larger signals (deeper red) represent a higher accessibility prediction on altered sequence, lower signals (deeper blue) represent lower accessibility on altered sequence. (**C**) Zoom in in silico nucleotide mutagenesis within region chr6: 43858285-43858494 of RPE cell type. Lower accessibility predicted on the enhancer with risk variant rs943079 A>G,T. (**D**) Zoom in in silico nucleotide mutagenesis within region chr6: 43858885-43858984 of RPE cell type. Higher accessibility predicted on the enhancer with risk variant rs943080 C>A. (**F**) In silico nucleotide mutagenesis influenced the prediction of accessibility within region chr10: 122453720-122455906 (0-based) of RPE cell type. (**G**) Zoom in in silico nucleotide mutagenesis within region chr10: 122453720-122453769 of RPE cell type. Higher accessibility predicted on the enhancer near to risk variant rs2672600 G>A, C. (**H**) Zoom in in silico nucleotide mutagenesis within region chr10: 122455860-122455906 of RPE cell type. Lower accessibility predicted on the enhancer near to risk variant rs3750847 C>T.

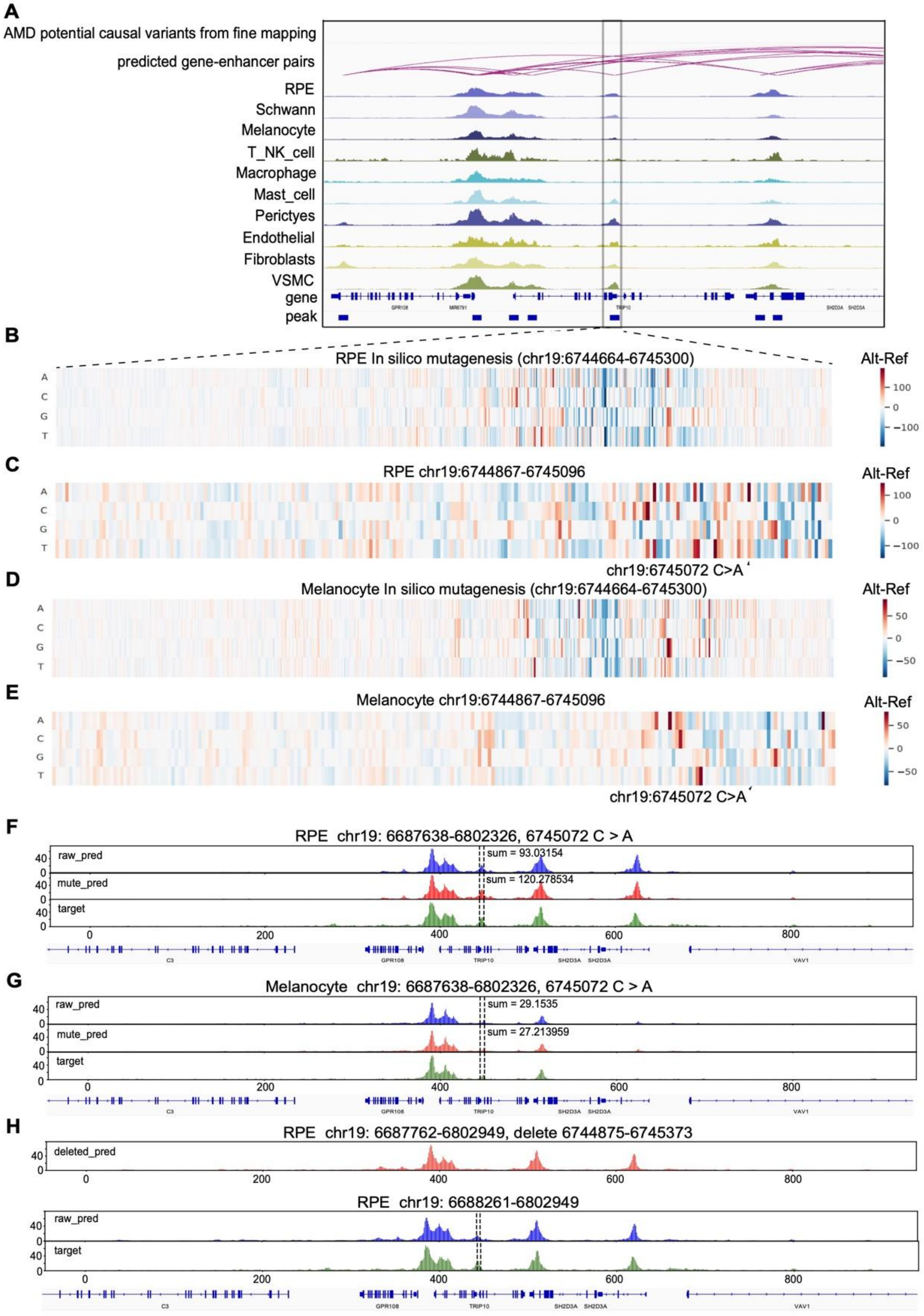
A
AMD potential causal variants from fine mapping
predicted gene-enhancer pairs
RPE
Schwann
Melanocyte
T_NK_cell
Macrophage
Mast_cell
Perictyes
Endothelial
Fibroblasts
VSMC
gene
peak
B
RPE In silico mutagenesis (chr19:6744664-6745300)
Alt-Ref
C
RPE chr19:6744867-6745096
chr19:6745072 C>A
D
Melanocyte In silico mutagenesis (chr19:6744664-6745300)
E
Melanocyte chr19:6744867-6745096
chr19:6745072 C>A
F
RPE chr19: 6687638-6802326, 6745072 C > A
raw_pred
sum = 93.03154
mute_pred
sum = 120.278534
target
G
Melanocyte chr19: 6687638-6802326, 6745072 C > A
raw_pred
sum = 29.1535
mute_pred
sum = 27.213959
target
H
RPE chr19: 6687762-6802949, delete 6744875-6745373
deleted_pred
RPE chr19: 6688261-6802949
raw_pred
target
C3
GPR108
TRIP10
SH2D3A
VAV1

Fig. S5. The Deep learning model helps to interpret mutation effects on noncoding risk variants of ocular disorder and traits for different cell types. (**A**) Fine mapping of AMD trait and causal risk variants in different categories of cCREs from cell types of RPE tissue. Genome browser tracks (GRCh38) display chromatin accessibility profiles from snATAC-seq, magenta arcs represent the predicted pairs of enhancer and gene from ABC model in RPE cell type. (**B** and **D**) In silico nucleotide mutagenesis influenced the prediction of accessibility within region chr19: 6744664-6745300 (0-based) of RPE cell type (B) and Melanocyte (D). Larger signals (deeper red) represent a higher accessibility prediction on altered sequence, lower signals (deeper blue) represent lower accessibility on altered sequence. (**C** and **E**) Zoom in in silico nucleotide mutagenesis within region chr19: 6744867-6745096 of RPE cell type (C) and Melanocyte (E). Higher accessibility predicted on the *TRIP10* enhancer with potential variant loci chr19 6745072 C>A for RPE and lower accessibility for Melanocyte. (**F** and **G**) Chromatin accessibility at *TRIP10* enhancer loci predicted in human RPE cell type (F) and Melanocyte (G). Green represents the raw target track, blue represents raw predicted track, red represents predicted track after mutation. (**H**) Chromatin accessibility after deleting TRIP10 enhancer loci predicted in human RPE cell type. Red represents predicted track after deletion mutation.

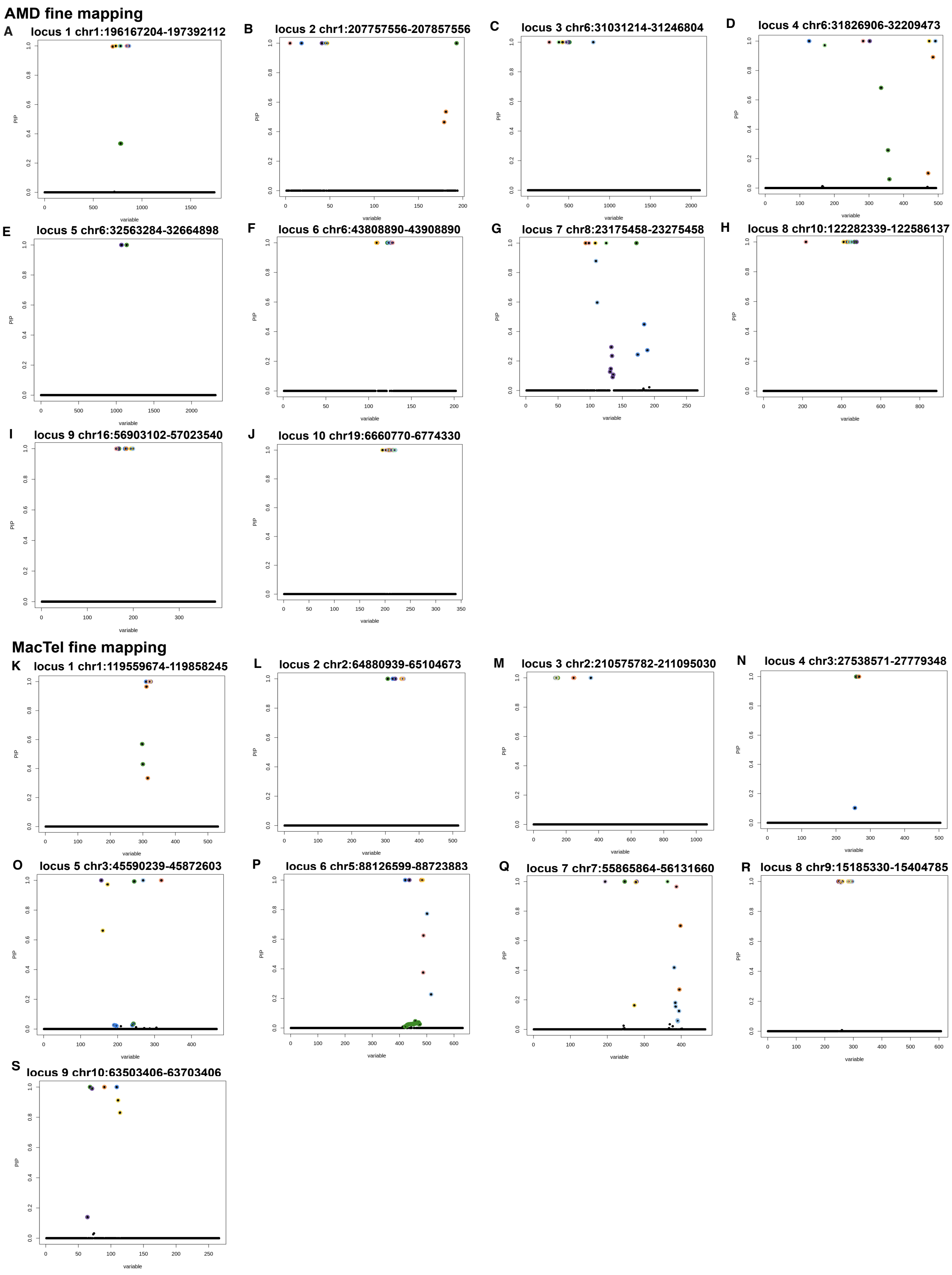
AMD fine mapping
A locus 1 chr1:196167204-197392112
B locus 2 chr1:207757556-207857556
C locus 3 chr6:31031214-31246804
D locus 4 chr6:31826906-32209473
E locus 5 chr6:32563284-32664898
F locus 6 chr6:43808890-43908890
G locus 7 chr8:23175458-23275458
H locus 8 chr10:122282339-122586137
I locus 9 chr16:56903102-57023540
J locus 10 chr19:6660770-6774330
MacTel fine mapping
K locus 1 chr1:119559674-119858245
L locus 2 chr2:64880939-65104673
M locus 3 chr2:210575782-211095030
N locus 4 chr3:27538571-27779348
O locus 5 chr3:45590239-45872603
P locus 6 chr5:88126599-88723883
Q locus 7 chr7:55865864-56131660
R locus 8 chr9:15185330-15404785
S locus 9 chr10:63503406-63703406
PIP
variable

Fig. S6. Loci of AMD trait and MacTel trait from fine mapping. (**A-J**) 10 found loci of AMD trait from fine mapping. PIP: Posterior Inclusion Probability. Colored spots represent causal SNPs in the region. (**K-S**) 9 found loci of MacTel trait from fine mapping.

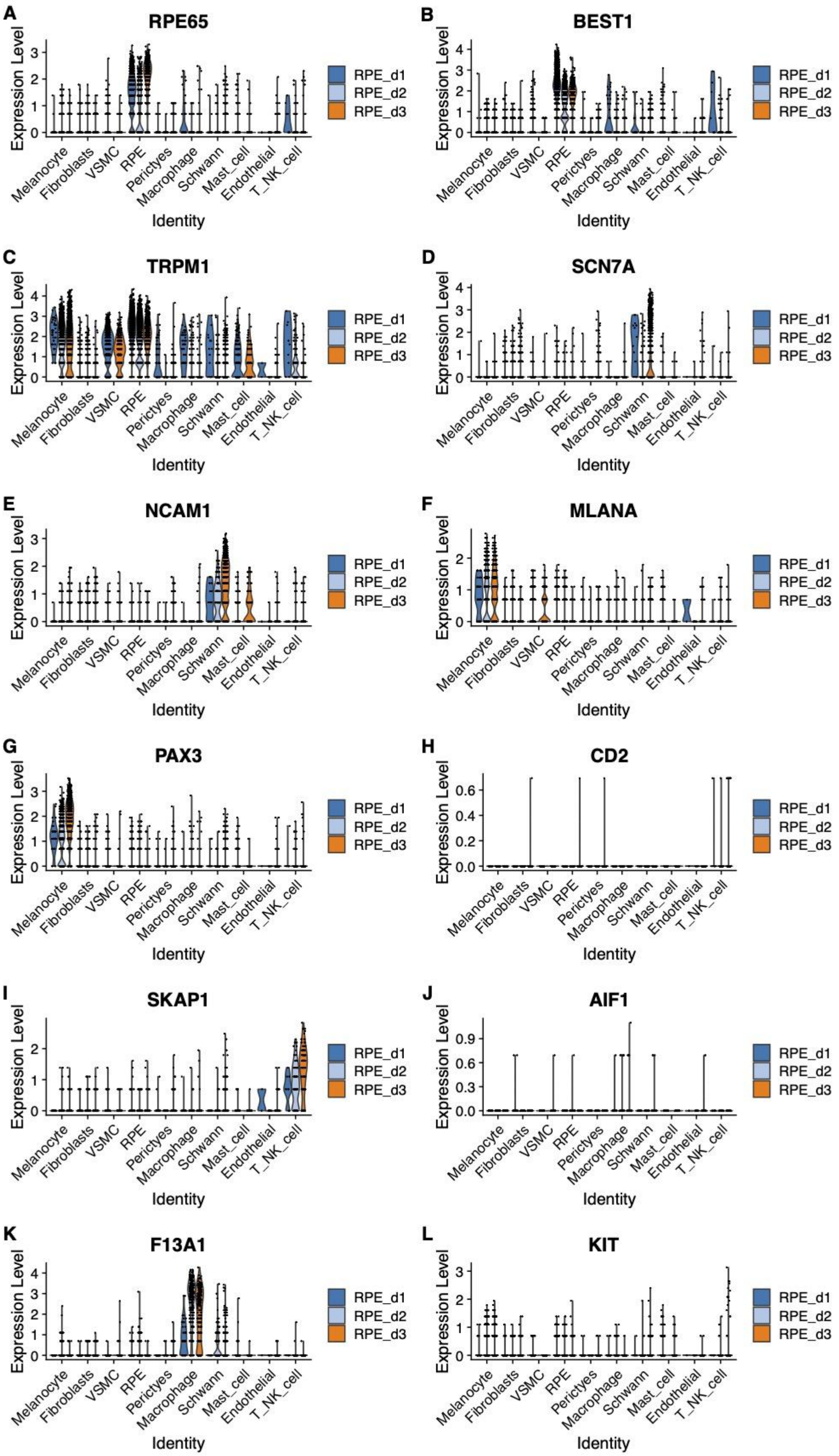
A
RPE65
B
BEST1
C
TRPM1
D
SCN7A
E
NCAM1
F
MLANA
G
PAX3
H
CD2
I
SKAP1
J
AIF1
K
F13A1
L
KIT
Expression Level
Identity
Melanocyte
Fibroblasts
VSMC
RPE
Perictyes
Macrophage
Schwann
Mast_cell
Endothelial
T_NK_cell
RPE_d1
RPE_d2
RPE_d3

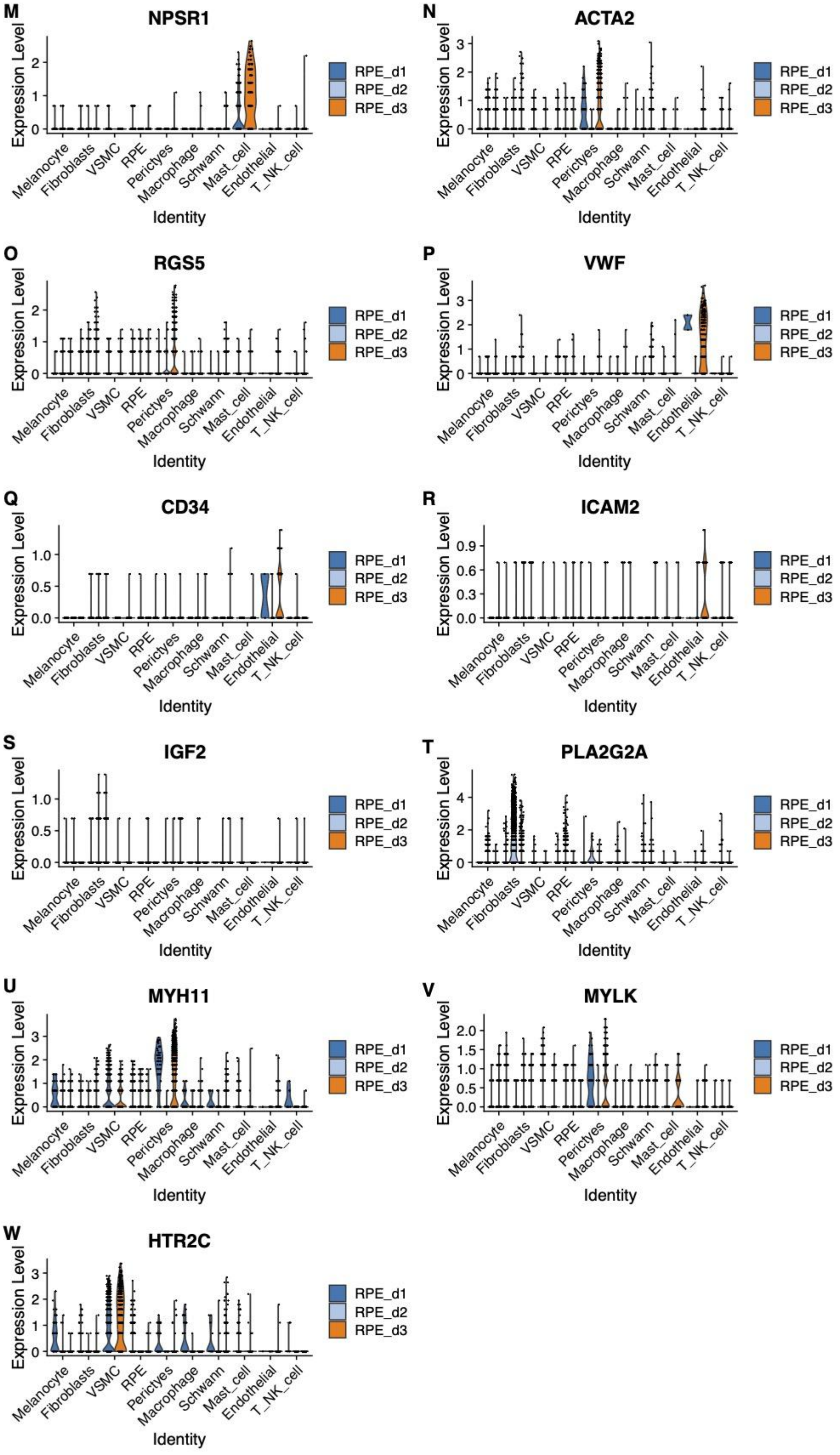

M
NPSR1
Expression Level
0
1
2
Melanocyte
Fibroblasts
VSMC
RPE
Perictyes
Macrophage
Schwann
Mast_cell
Endothelial
T_NK_cell
Identity
RPE_d1
RPE_d2
RPE_d3
N
ACTA2
Expression Level
0
1
2
3
Melanocyte
Fibroblasts
VSMC
RPE
Perictyes
Macrophage
Schwann
Mast_cell
Endothelial
T_NK_cell
Identity
RPE_d1
RPE_d2
RPE_d3
O
RGS5
Expression Level
0
1
2
Melanocyte
Fibroblasts
VSMC
RPE
Perictyes
Macrophage
Schwann
Mast_cell
Endothelial
T_NK_cell
Identity
RPE_d1
RPE_d2
RPE_d3
P
VWF
Expression Level
0
1
2
3
Melanocyte
Fibroblasts
VSMC
RPE
Perictyes
Macrophage
Schwann
Mast_cell
Endothelial
T_NK_cell
Identity
RPE_d1
RPE_d2
RPE_d3
Q
CD34
Expression Level
0.0
0.5
1.0
Melanocyte
Fibroblasts
VSMC
RPE
Perictyes
Macrophage
Schwann
Mast_cell
Endothelial
T_NK_cell
Identity
RPE_d1
RPE_d2
RPE_d3
R
ICAM2
Expression Level
0.0
0.3
0.6
0.9
Melanocyte
Fibroblasts
VSMC
RPE
Perictyes
Macrophage
Schwann
Mast_cell
Endothelial
T_NK_cell
Identity
RPE_d1
RPE_d2
RPE_d3
S
IGF2
Expression Level
0.0
0.5
1.0
Melanocyte
Fibroblasts
VSMC
RPE
Perictyes
Macrophage
Schwann
Mast_cell
Endothelial
T_NK_cell
Identity
RPE_d1
RPE_d2
RPE_d3
T
PLA2G2A
Expression Level
0
2
4
Melanocyte
Fibroblasts
VSMC
RPE
Perictyes
Macrophage
Schwann
Mast_cell
Endothelial
T_NK_cell
Identity
RPE_d1
RPE_d2
RPE_d3
U
MYH11
Expression Level
0
1
2
3
Melanocyte
Fibroblasts
VSMC
RPE
Perictyes
Macrophage
Schwann
Mast_cell
Endothelial
T_NK_cell
Identity
RPE_d1
RPE_d2
RPE_d3
V
MYLK
Expression Level
0.0
0.5
1.0
1.5
2.0
Melanocyte
Fibroblasts
VSMC
RPE
Perictyes
Macrophage
Schwann
Mast_cell
Endothelial
T_NK_cell
Identity
RPE_d1
RPE_d2
RPE_d3
W
HTR2C
Expression Level
0
1
2
3
Melanocyte
Fibroblasts
VSMC
RPE
Perictyes
Macrophage
Schwann
Mast_cell
Endothelial
T_NK_cell
Identity
RPE_d1
RPE_d2
RPE_d3

Fig. S7. Marker gene expression level across different donors for each cell type in RPE/choroid tissue. **(A)** Marker gene RPE65 **(B)** BEST1 **(C)** TRPM1 **(D)** SCN7A **(E)** NCAM1 **(F)** MLANA **(G)** PAX3 **(H)** CD2 **(I)** SKAP1 **(J)** AIF1 **(K)** F13A1 **(L)** KIT **(M)** NPSR1 **(N)** ACTA2 **(O)** RGS5 **(P)** VWF **(Q)** CD34 **(R)** ICAM2 **(S)** IGF2 **(T)** PLA2G2A **(U)** MYH11 **(V)** MYLK **(W)** HTR2C. The values of gene expression were normalized with function SCTransform() of Seurat, the expression level shows the normalized results of gene expression for better visualization and comparison.

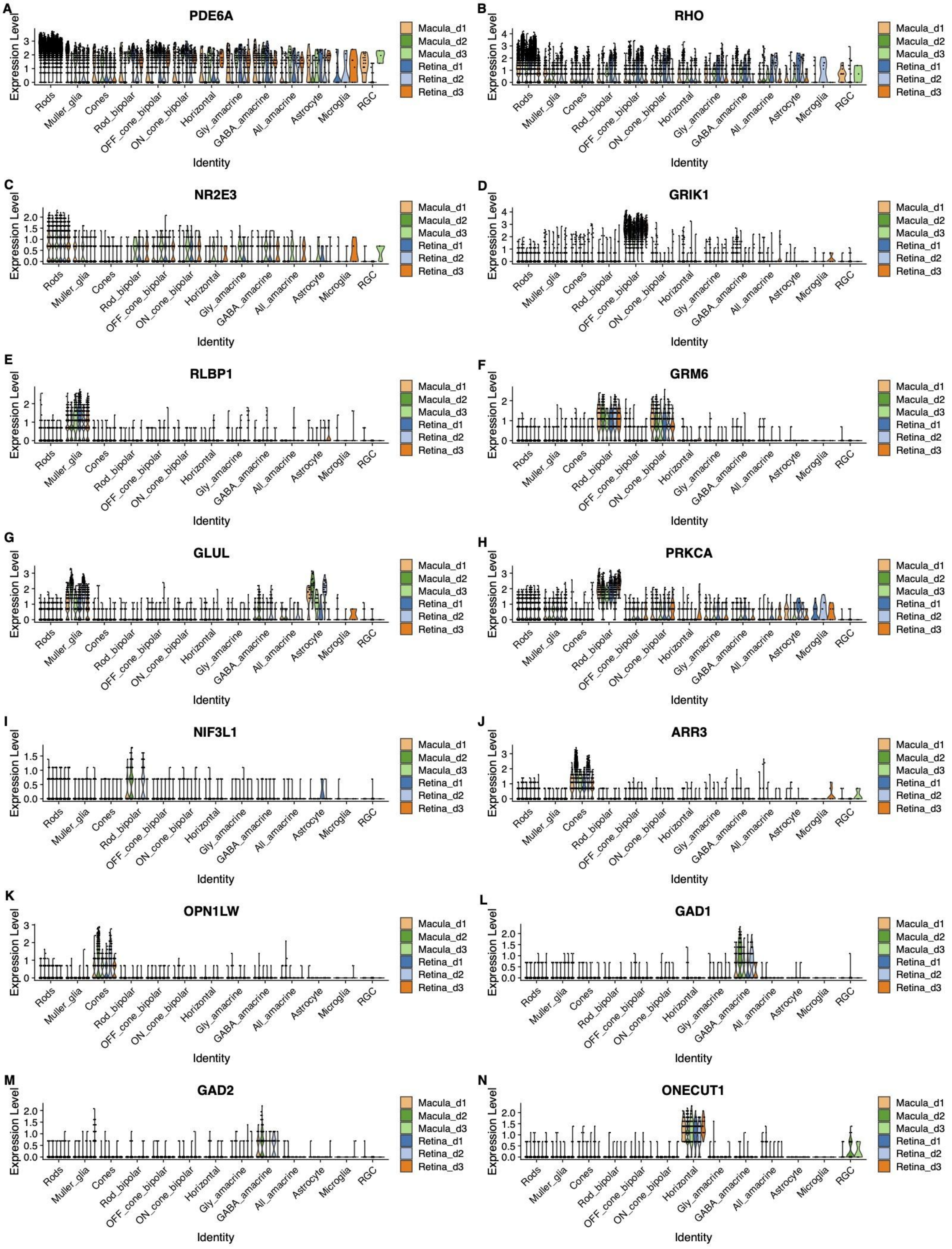
A
PDE6A
B
RHO
C
NR2E3
D
GRIK1
E
RLBP1
F
GRM6
G
GLUL
H
PRKCA
I
NIF3L1
J
ARR3
K
OPN1LW
L
GAD1
M
GAD2
N
ONECUT1
Expression Level
Identity
Rods
Muller_glia
Cones
Rod_bipolar
OFF_cone_bipolar
ON_cone_bipolar
Horizontal
Gly_amacrine
GABA_amacrine
AII_amacrine
Astrocyte
Microglia
RGC
Macula_d1
Macula_d2
Macula_d3
Retina_d1
Retina_d2
Retina_d3

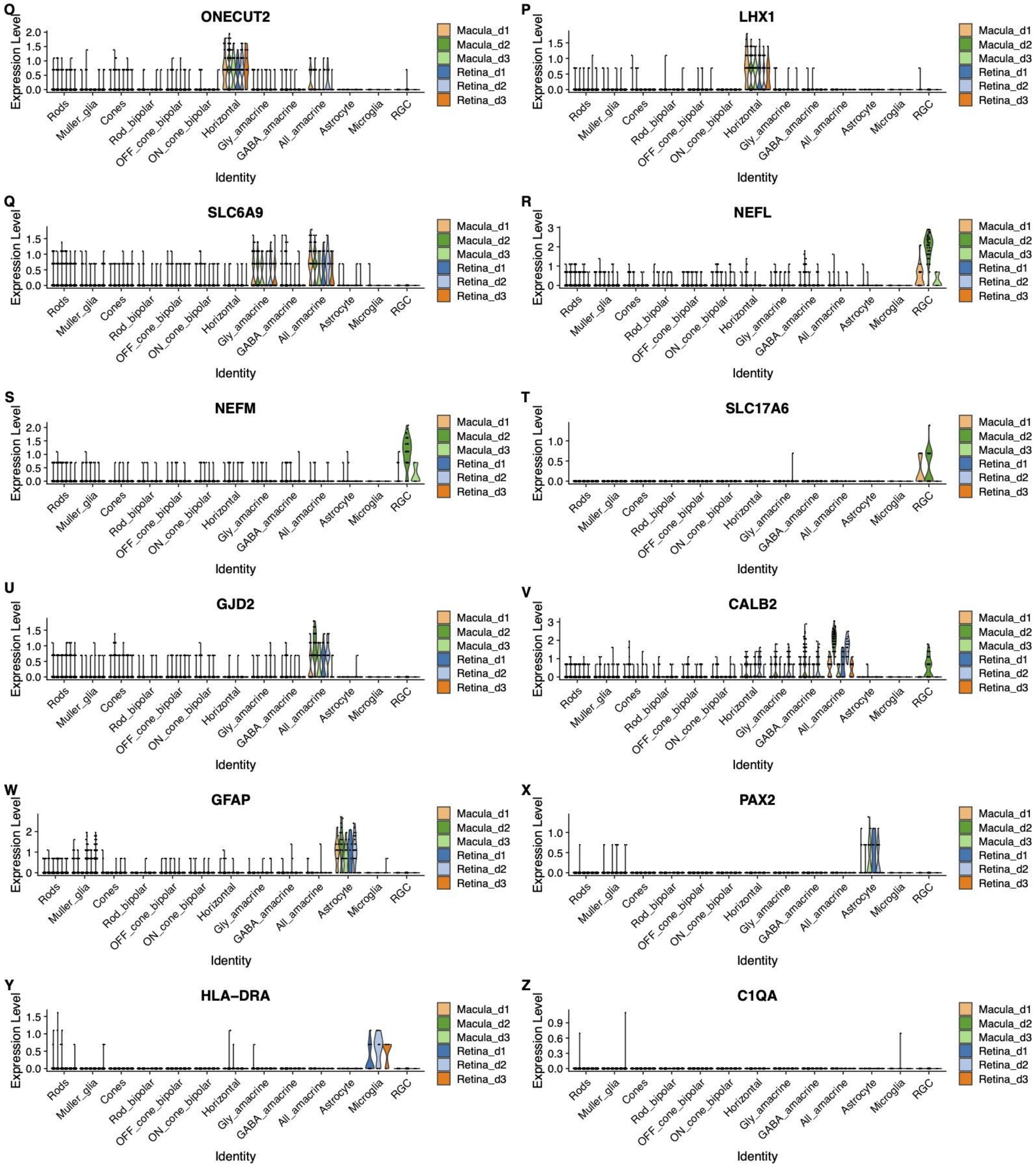

Fig. S8. Marker gene expression level across different donors for each cell type in retina and macula tissue. **(A)** Marker gene PDE6A **(B)** RHO **(C)** NR2E3 **(D)** GRIK1 **(E)** RLBP1 **(F)** GRM6 **(G)** GLUL **(H)** PRKCA **(I)** NIF3L1 **(J)** ARR3 **(K)** OPN1LW **(L)** GAD1 **(M)** GAD2 **(N)** ONECUT1 **(O)** ONECUT2 **(P)** LHX1 **(Q)** SLC6A9 **(R)** NEFL **(S)** NEFM **(T)** SLC17A6 **(U)** GJD2 **(V)** CALB2 **(W)** GFAP **(X)** PAX2 **(Y)** HLA-DRA **(Z)** C1QA. The values of gene expression were normalized with function SCTransform() of Seurat, the expression level shows the normalized results of gene expression for better visualization and comparison.

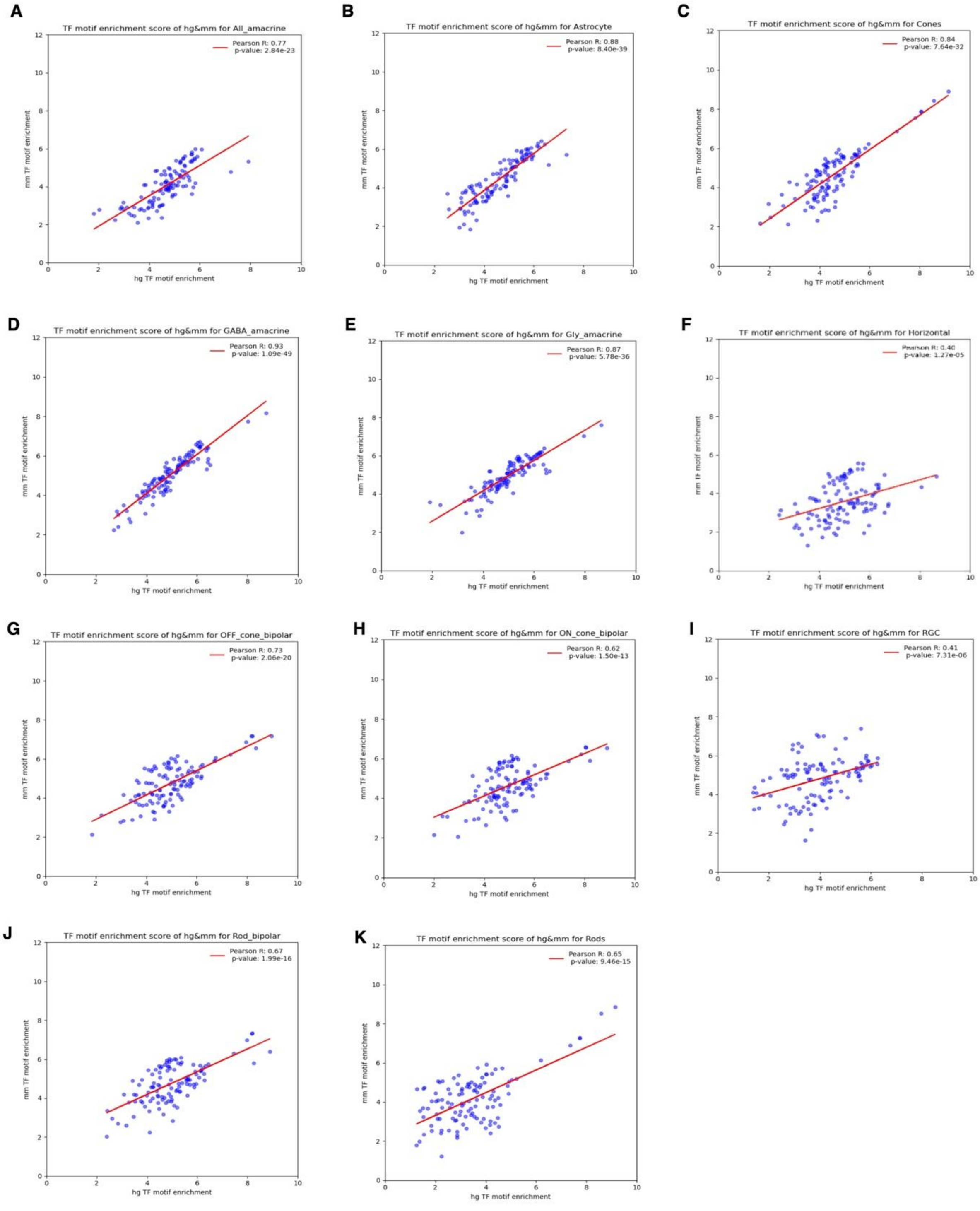

Fig. S9. The conserved TF motifs of human and mouse retina for each cell type and the PCC of the enrichment score. (**A**) All amacrine **(B)** Astrocyte **(C)** Cones **(D)** GABA amacrine **(E)** Gly amacrine **(F)** Horizontal **(G)** OFF cone bipolar **(H)** ON cone bipolar **(I)** RGC **(J)** Rod bipolar **(K)** Rods.

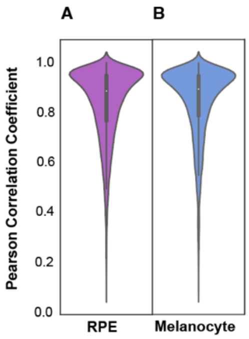

Fig. S10. The violin plot of Pearson Correlation Coefficient between deep learning model predicted open chromatin in RPE and melanocytes and psuedobulk ATAC-seq data for each cell type on its validation dataset. (**A**) Pseudo bulk RPE **(B)** Pseudo bulk Melanocyte.

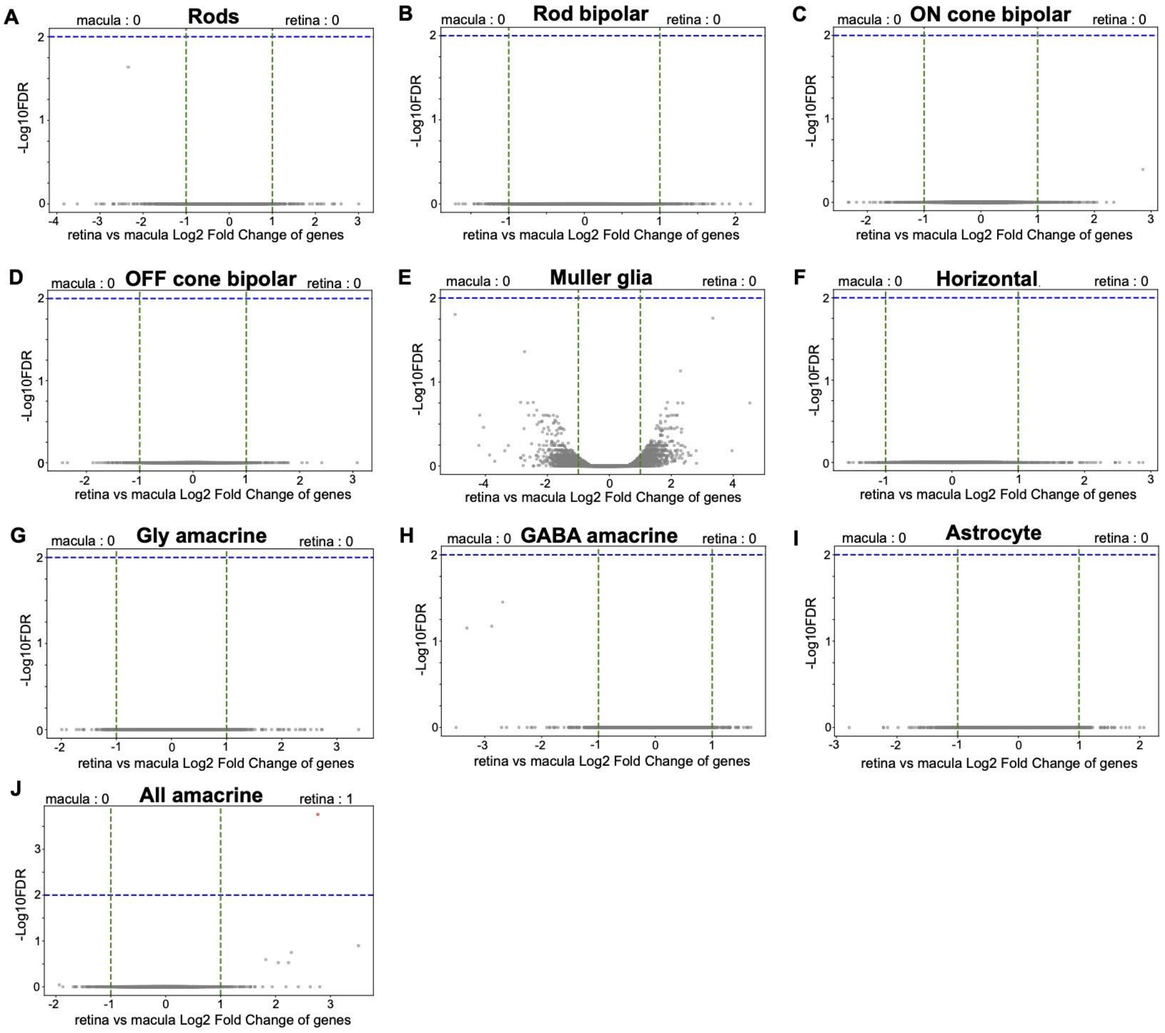

Fig. S11. Differential gene expression between human retina and macula. (**A**) Pseudo bulk Rods **(B)** Pseudo bulk Rod bipolar **(C)** Pseudo bulk ON cone bipolar **(D)** Pseudo bulk OFF cone bipolar **(E)** Pseudo bulk Muller glia **(F)** Pseudo bulk Horizontal **(G)** Pseudo bulk Gly amacrine **(H)** Pseudo bulk GABA amacrine **(I)** Pseudo bulk Astrocyte **(J)** Pseudo bulk All amacrine

Other Supplementary Materials for this manuscript includes:

Tables S1 to S15

Table S1: Meta data table for all the samples

Table S2: Known marker gene table for annotation of human retina & macula cell types

Table S3: Cell type specific genes table of human retina & macula cell types

Table S4: Known marker gene table for annotation of human RPE/choroid cell types

Table S5: Cell type specific genes table of human RPE/choroid cell types

Table S6: Cell type specific cCREs table of human retina & macula cell types

Table S7: Cell type specific cCREs table of human RPE/choroid cell types

Table S8: Cell type specific TF motifs table of human retina & macula cell types

Table S9: Cell type specific TF motifs table of human RPE/choroid cell types

Table S10: Table of cCREs of peak-gene pairs from ABC model prediction for human retina & macula cell types

Table S11: Table of genes of peak-gene pairs from ABC model prediction for human retina & macula cell types

Table S12: Table of cCREs of peak-gene pairs from ABC model prediction for human RPE/choroid cell types

Table S13: Table of genes of peak-gene pairs from ABC model prediction for human RPE/choroid cell types

Table S14: Known marker gene table for annotation of mouse retina cell types

Table S15: Cell type specific TF motifs table of mouse retina cell types
